# Transparency and information sharing could help abate the COVID-19 pandemic

**DOI:** 10.1017/ice.2020.174

**Published:** 2020-04-22

**Authors:** Farid Rahimi, Amin Talebi Bezmin Abadi

**Affiliations:** 1 Research School of Biology, The Australian National University, Canberra, Australia; 2 Department of Bacteriology, Faculty of Medical Sciences, Tarbiat Modares University, Tehran, Iran

*To the Editor*—In December 2019, a surge of patients with a pneumonia-like illness in Wuhan (Hubei Province, China) foreshadowed the outbreak of a new disease. Thereafter, the highly contagious nature of the virus and the rapid spread of the outbreak attracted global attention and caused apprehension.^1^ The causative agent of the disease was recognized and labeled severe acute respiratory syndrome coronavirus 2 (SARS-CoV-2), and the disease was named coronavirus disease 2019 (COVID-19). SARS-CoV-2 moved across the Chinese borders within a month and is now a pandemic that seriously threatens global public health.^2^ Shortly after China’s status as the COVID-19 epicentral locus, Italy and the United States suffered high numbers of infected cases, with the 2 highest fatality counts.^3-5^ The rapid viral spread globally has confirmed the devastating nature of SARS-CoV-2 and its tendency to gravely affect vulnerable individuals of older age with preexisting conditions including chronic obstructive pulmonary disease, cardiovascular disease, and diabetes. According to Worldometer, 126,066 people had died due to COVID-19 and 1,992,189 positive cases have been confirmed as of April 14, 2020.^5^

Some putative, unproven traditional or homemade therapies have been suggested to alleviate the severe COVID-19 symptoms or claimed to treat them.^6-8^ Unfortunately, a universally approved or scientifically proven vaccine or drug to prevent or treat COVID-19 does not currently exist.^9^ Consequently, therapeutic interventions for managing severe cases are limited only to respiratory support by ventilation or extracorporeal membrane oxygenation.^9^ COVID-19 can also present as a mild disease in young adults or in children, but anyone can be affected, with various outcomes. So far, countermeasures to control the pandemic include self-isolation, physical distancing, strict hygiene, cough and sneeze etiquette, and quarantine. However, compliance with such countermeasures are particularly abysmal in developing countries because of limited governmental resources or oversight, lack of surveillance systems, general poverty, or inaccessible information. In many developing or developed countries, poorly resourced or unprepared healthcare systems have hampered urgent and decisive responses against the pandemic due to lack of insufficient testing, unsatisfactory case finding or tracing, underresourced intensive care, and an overstretched healthcare workforce. Here, we briefly highlight the importance of public education and information sharing in addressing the pandemic and encouraging public compliance.

Transparent and accurate information sharing nationally and globally are important in the fight against SARS-CoV-2. For example, Italy adopted a transparent strategy following registration of the first COVID-19 case^10^; the aim was to avoid unreasonable public panic and confusion through media or other channels. The World Health Organization has been providing daily updates and situation reports on the progression of the pandemic and has provided global guidance and support.

The economical and psychosocial effects of the pandemic have already been profound enough to have affected or shattered the social fabrics in some countries. Public panic due to lack of awareness could result in unrest, instability, and potentially, a disaster that will be difficult to control. Thus, all governments or nations should boost public awareness campaigns about the virus, about the disease and pandemic and about how best to achieve individual protection from viral exposure. Unprotected exposure to hospitalized patients, confirmed cases in self-isolation, or suspected carriers must be avoided. Although SARS-CoV-2 has inevitably caused high levels of public tribulation, taking reassuring actions and maintaining everyday provisions and some basic protection (eg, face masks) are steps that have met some of the public’s expectations and have potentially prevented panic. Transparency and clear governmental and national guidance and coordination on how to manage the pandemic is of utmost importance to avoid public confusion and, importantly, to encourage or otherwise enforce compliance.

In conclusion, the mandated nationwide restrictions and quarantine regimens in different countries have been timely and represent a uniformity of countermeasures that will abate the pandemic’s impacts. Furthermore, solidarity against the virus calls for globally united and coordinated actions to efficiently control the outbreak. Although we recognize that the circumstances and cultural or social fabrics of each nation are unique and that governmental responses may differ to accommodate unique circumstances, solidarity, transparency, and rapid data sharing nationally and internationally must discount political, regional, economical, religious, and racial differences in the fight against this nondiscriminatory, but common, viral enemy.
